# Supplementary material for: A Resource-Virtualized and Hardware-Aware Quantum Compilation Framework for Real Quantum Computing Processors
Source: Research (Wash D C). 2025 Oct 16;8:0947. doi: 10.34133/research.0947 (PMC12528855; doi:10.34133/research.0947)
Supplement: Supplementary 1 — Sections S1 to S5 Figs. S1 to S11 [file research.0947.f1.pdf]

Supplementary Materials for "A Resource-Virtualized and Hardware-Aware Quantum Compilation Framework for Real Quantum Computing Processors"

September 10, 2025

Contents

S1 Interfaces and Use Cases **2**
S1.1 Backend Interface . . . 2
S1.2 Compiler Interface . . . 2
S1.3 Use Cases of Transpiler . . . 3

S2 Complexity Analysis of VQPUs Creation **4**
S2.1 Theoretical analysis . . . 4
S2.2 Numerical Simulation . . . 5

S3 Weisfeiler-Lehman (WL) subtree kernel algorithm **6**

S4 Components of the pass module **7**
S4.1 Decomposition . . . 8
S4.2 Unroll . . . 8
S4.3 Optimization . . . 8
S4.4 Parameter Tuning . . . 9

S5 Performance Comparisons **9**
S5.1 Experimental Results and Fidelity–Cost Relationship . . . 12
S5.2 Simulation results . . . 12
S5.3 System Stability under Hardware Calibration Dynamics . . . 12

S1 Interfaces and Use Cases

To facilitate the deployment of QSteed on quantum hardware or quantum clusters, we provide two primary interfaces: one for backend hardware, enabling quantum resource management, and the other for frontend quantum tasks, supporting quantum compilation.

S1.1 Backend Interface

To facilitate the updating and addition of quantum computing resource information, QSteed provides a unified interface for quantum computing resource virtualization management, `update_chip_api`. By specifying the quantum chip name, `chip_name`, and a dictionary containing the quantum chip information, `chip_info_dict`, users can easily add or update quantum chip information in the quantum resource virtualization database. A specific usage example is shown below:

```
from qsteed.apis.resourceDB_api import update_chip_api
import json
chip_name = "example"
chip_file = "chipexample.json"
with open(chip_file, "r") as file:
    chip_info_dict = json.load(file)
update_chip_api(chip_name, chip_info_dict)
```

Comprehensive usage examples and deployment instructions for the underlying database can be found on the project's open-source GitHub repository [1].

S1.2 Compiler Interface

As middleware, QSteed provides the quantum compiler interface, `call_compiler_api`. Through this interface, user tasks can be sent to the quantum compiler, which returns the compilation information along with the compiled QASM circuit upon completion. The compiled QASM can then be sent to the control system for executing quantum computing tasks. A specific usage example is provided below:

```
from qsteed.apis.compiler_api import call_compiler_api

# Assume you can obtain the user's task information and store it as task_info.
task_info = {
    "circuit": qasm,   # user's quantum circuit
    "transpile": True, # True-Perform transpilation
                       # False-Only program verification
    "qpu_name": "example", # If none, automatically selected
    "qubits_list": None, # Specify the list of qubits to use
    "optimization_level": 2, # Preset optimization (0-3)
    "passflow": None, # Custom transpilation Passflow
    "vqpu_preferred": "fidelity" # VQPU selector preference, "fidelity" or "structure"
}
compiled_results = call_compiler_api(**task_info)
compiled_QASM = compiled_results[0]
```

```python
qubits_to_cbits = compiled_results[1]
compiled_info = compiled_results[2]
```

S1.3 Use Cases of Transpiler

If you are interested in exploring quantum compilation algorithms with QSteed without the need for deployment on quantum hardware, you can focus solely on the quantum transpiler module within QSteed. By customizing the quantum backend, initial model, and passflow, you can use the `Transpiler` to perform quantum circuit transpilation. The following is an example of the implementation:

```python
from qsteed import *

rqc = RandomCircuit(num_qubit=5, gates_number=30)
qc = rqc.random_circuit()

# Backend and initial model settings
basis_gates = ['cx', 'rx', 'ry', 'rz']
coupling_list = [(0, 1, 0.991), (1, 2, 0.976), (2, 3, 0.985), (2, 4, 0.994)]
backend_properties = {
    "name": "ExampleBackend",
    "backend_type": "superconducting",
    "qubits_num": 5,
    "coupling_list": coupling_list,
    "basis_gates": basis_gates,
}
backend_instance = Backend(**backend_properties)
initial_model = Model(backend=backend_instance)

# Predefined transpilation passflow
passes = [
    UnrollTo2Qubit(),
    SabreLayout(heuristic='mixture', max_iterations=3),
    UnrollToBasis(basis_gates=basis_gates),
    GateCombineOptimization(),
    OneQubitGateOptimization(),
    ParaSubstitution()
]  # You can also add your custom pass here.
passflow = PassFlow(passes=passes)

# Perform quantum transpilation
transpiler = Transpiler(passflow, initial_model)
transpiled_circuit = transpiler.transpile(qc)
```

Additionally, to facilitate the examination of each pass's execution efficiency and the changes in quantum circuits during the transpilation process, QSteed provides a visualization module for transpilation intermediate results, `TranspilerVis`. This module adopts a three-stage design: (1) During the transpilation process, it collects real-time execution data for each pass, such as gate

counts, circuit depth, and execution time. (2) The collected data is organized into two levels of information structure: detailed and summarized. (3) An interactive interface is then used to provide multidimensional analytical views, including statistical tables, circuit comparisons, model viewing, etc.

This design not only makes the compilation process more transparent and controllable, aiding developers in debugging and optimizing compilation strategies, but also provides intuitive data visualization to help understand the role and performance characteristics of each pass. This is of significant value in enhancing the efficiency of quantum transpiler development and optimization. An example implementation is shown below:

```python
from qsteed.transpiler.transpiler_visualization import TranspilerVis, dynamic_draw

transpiler_vis = TranspilerVis(passflow, initial_model)
transpiled_circuit, info, short_info = transpiler_vis.transpile_vis(qc)
dynamic_draw(info, short_info)
```

Pass	Pass Name	Execution Time (ms)	Total Gates Before	Total Gates After	2qubit Gates Before	2qubit Gates After	Depth Before	Depth After	Layout changed status
Pass_0	UnrollTo2Qubit	0.37	21	53	1	17	14	31	False
Pass_1	SabreLayout	16.46	53	57	17	21	31	29	True
Pass_2	UnrollToBasis	1.24	57	71	21	30	29	37	False
Pass_3	GateCombineOptimization	2.61	71	64	30	28	37	37	False
Pass_4	OneQubitGateOptimization	2.84	64	60	28	28	37	36	False

Figure S1: A visualization of the results from a circuit compilation process.

Figure S1 illustrates the visualized results of a circuit compilation process. It allows users to easily observe which compilation pass is most dominant and which is the most time-consuming, thereby facilitating targeted performance optimization in future developments.

S2 Complexity Analysis of VQPUs Creation

S2.1 Theoretical analysis

In this subsection, we provide a detailed analysis of the time complexity of the heuristic Algorithm 1 for identifying optimal substructures of the chip.

Let the number of qubits in the chip be N, the number of coupling edges be M, and the maximum degree be Δ. During the initialization stage (lines 1–6), sorting the N nodes and M edges by fidelity requires $O(N \log N)$ and $O(M \log M)$ time, respectively, while the remaining operations are constant-time, yielding a total cost of $O(N \log N + M \log M)$.

The main loop (lines 7–21) contains three nested loops: (i) the outer loop iterates over target sizes $n = 3, \ldots, N$; (ii) the method loop iterates over a constant number of strategies; and (iii) the

edge loop scans all M seed edges. Inside the *while* loop, the subgraph grows from 2 nodes to n nodes using a priority-queue–based procedure. In each step of the *while* loop, the set of candidate neighbors has size at most $B(n) = \min\{\Delta n, N\}$. Extracting the optimal neighbor from the priority queue requires $O(\log B(n))$, so after $n-2$ iterations the overall cost is $O(n \log B(n))$. In addition, upon generating all candidate subgraphs of size n (approximately $3M$ candidates), the algorithm sorts them by average fidelity for selection (line 20), introducing an extra cost of $O(M \log M)$ for each n. Therefore, the per-n complexity is

$$T_{\text{per-}n} = O(M\, n \log B(n)) + O(M \log M).$$

Summing over $n = 3$ to N, we obtain

$$T_{\text{main}} = \sum_{n=3}^{N} O(M\, n \log B(n)) + \sum_{n=3}^{N} O(M \log M).$$

The total time complexity of the algorithm is thus

$$T_{\text{total}} = T_{\text{main}} + O(N \log N + M \log M).$$

For sparse coupling graphs ($\Delta = O(1)$, as commonly found in superconducting chips), we have $B = \Delta n = O(n)$, then,

$$T_{\text{total}} = O(MN^2 \log N) + O(NM \log M) + O(N \log N + M \log M).$$

In this case, the first term is dominant, and the overall time complexity can be expressed as

$$T_{\text{total}} = O(MN^2 \log N).$$

For sparsely coupled superconducting chips, we further have $M = O(N)$, and the complexity simplifies to

$$T_{\text{total}} = O(N^3 \log N).$$

S2.2 Numerical Simulation

This section presents the numerical results of the complexity analysis. We consider three chip topologies—square lattice,heavy-hexagonal, and hexagonal—as illustrated in Figure S2. For each structure, we plot the variation of the number of VQPUs, the time required to identify substructures, and the update time of the resource virtualization database as the number of physical qubits increases (see Figure S3, S4, and S5). It can be observed that the time required by Algorithm 1 for identifying the optimal substructures scales as $O(N^3 \log N)$, which is consistent with the theoretical analysis. The current bottleneck lies in the database update and write operations, with the fitted runtime scaling approximately as $O(N^4)$. The primary reason is that the present implementation uses MySQL with a serial procedure for deleting old records and inserting new ones. Future improvements

may include adopting a primary-key–based strategy of differential deletion and batch insertion, as well as redesigning the database architecture or migrating to more efficient systems such as MongoDB or Redis.

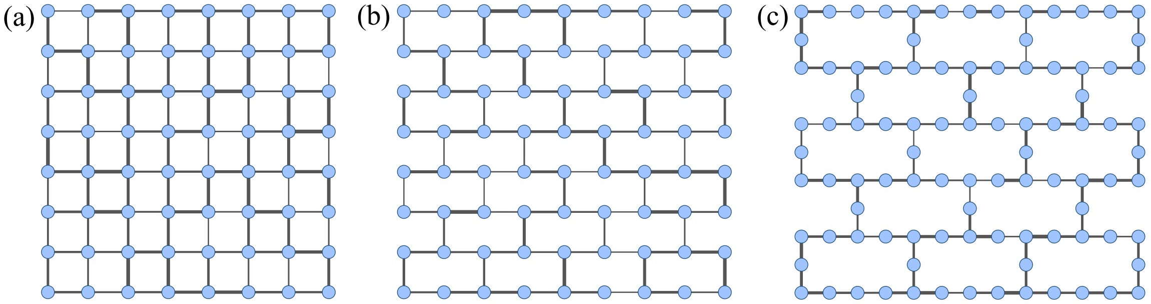

Figure S2: Chip topology structures: (a) square lattice, (b) hexagonal lattice, and (c) heavy-hexagonal lattice.

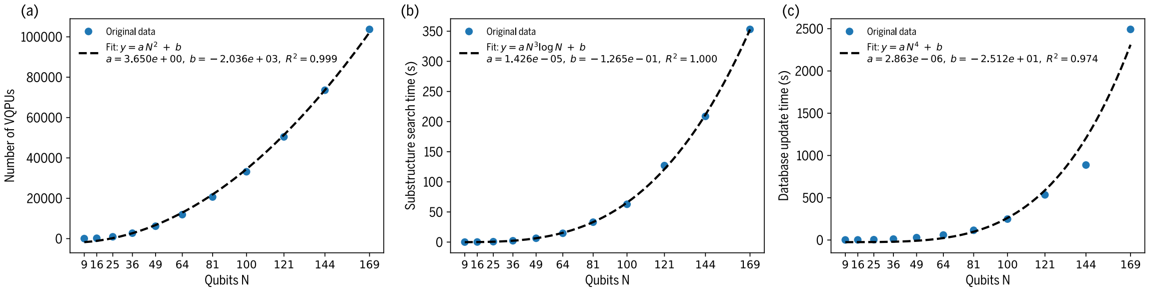

Figure S3: Chip with square lattice topology. Panels (a), (b), and (c) respectively show the variation of the number of VQPUs, the time required for substructure identification, and the update time of the resource virtualization database as the number of physical qubits N increases.

S3 Weisfeiler-Lehman (WL) subtree kernel algorithm

The WL subtree kernel measures the similarity between two graphs, G_1 and G_2, by iteratively updating node labels and computing kernel values. The overall kernel value is given by [2]:

$$K(G_1, G_2) = \sum_{t=1}^{T} k_t(G_1, G_2), \tag{S1}$$

where $k_t(G_1, G_2)$ represents the subtree kernel value at iteration t. At each iteration, this value is computed by comparing the edges of G_1 and G_2, considering both structural equivalence and, optionally, edge weights. The kernel at iteration t is defined as:

$$k_t(G_1, G_2) = \sum_{(u,v)\in E_1} \sum_{(x,y)\in E_2} \delta((u, v), (x, y)) \cdot f(w_{uv}, w_{xy}), \tag{S2}$$

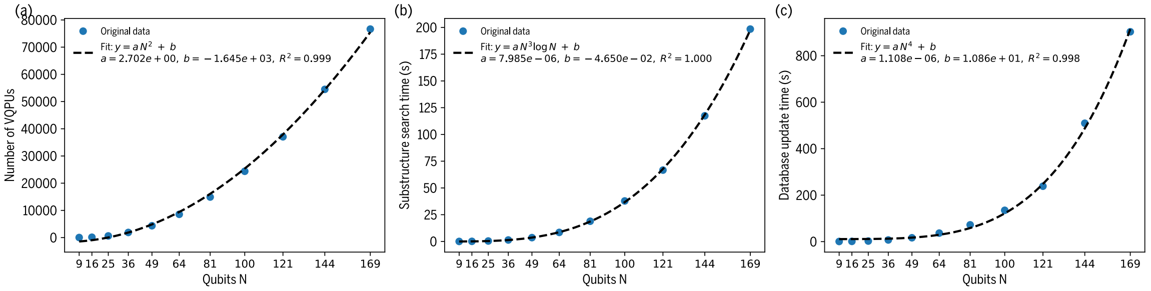

Figure S4: Chip with hexagonal lattice topology. Panels (a), (b), and (c) respectively show the variation of the number of VQPUs, the time required for substructure identification, and the update time of the resource virtualization database as the number of physical qubits N increases.

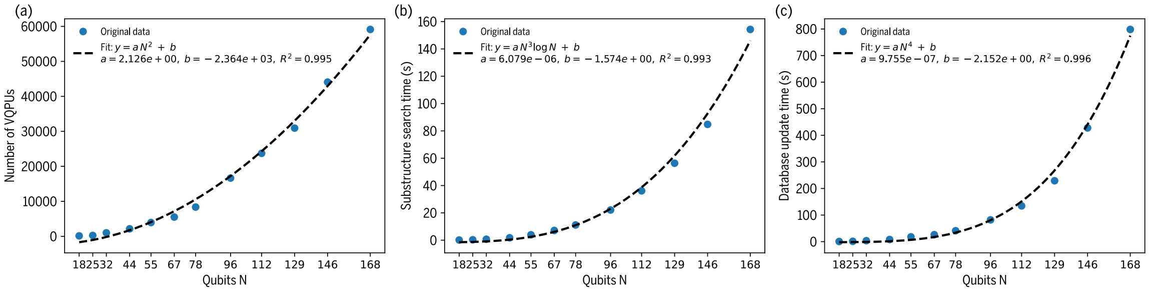

Figure S5: Chip with heavy-hexagonal lattice topology. Panels (a), (b), and (c) respectively show the variation of the number of VQPUs, the time required for substructure identification, and the update time of the resource virtualization database as the number of physical qubits N increases.

where $\delta((u,v),(x,y)) = 1$ if $u = x$ and $v = y$; otherwise, $\delta = 0$. The function $f(w_{uv}, w_{xy})$ evaluates as $\frac{1}{\exp((w_{uv}-w_{xy})^2)}$ when edge weights are considered, or $f(w_{uv}, w_{xy}) = 1$ otherwise. At the same time, node labels are updated by aggregating the current label of the node with the labels of its neighbors, generating a unique new label:

$$l_t(v) \leftarrow \text{unique}\left(l_{t-1}(v) \,||\, \text{sorted}(\{l_{t-1}(u) \mid u \in \mathcal{N}(v)\})\right), \tag{S3}$$

where $l_t(v)$ denotes the label of node v at iteration t, while unique represents a mapping function that ensures label uniqueness, and $||$ denotes the concatenation operator. A higher kernel value $K(G_1, G_2)$ indicates greater structural similarity between the two graphs. By computing the kernel values between the quantum circuit and all available VQPUs, the module identifies the VQPU with the highest structural resemblance. In cases where multiple VQPUs yield the same kernel value, the one with the highest overall fidelity is selected.

S4 Components of the pass module

The `Pass` module comprises multiple core components responsible for critical functions. These include the decomposition of multi-qubit gates, the expansion of equivalent quantum gates, qubit mapping and routing, quantum circuit optimization, and the tuning of parameters within parame-

terized quantum circuits. Our implementation of qubit mapping and routing was described in the main body of this paper. In this section, we provide a detailed exposition of the other aforementioned components.

S4.1 Decomposition

Numerous theoretical methods have been proposed for universal quantum gate decomposition, including QR decomposition [3], quantum Shannon decomposition [4], and cosine-sine decomposition (CSD) [5]. In addition, approximate quantum circuit synthesis algorithms have been shown to significantly reduce gate counts [6, 7]; however, their exponential computational complexity limits their practical scalability to small circuits involving only 2–5 qubits. Reinforcement learning–based approaches have also demonstrated substantial improvements in gate efficiency [8, 9], but their applicability remains largely constrained to 1–3 qubit gates due to the high cost of training.

This study does not aim to design new or more efficient synthesis techniques. Instead, to decompose an arbitrary n-qubit gate into a set of universal basic gates, the current version of QSteed adopts a combination of well-established techniques: ZYZ decomposition [10] for single-qubit operations, KAK decomposition [11] for two-qubit operations, and cosine-sine decomposition (CSD) [5] for general multi-qubit operations.

S4.2 Unroll

This component involves a series of equivalent transformations of quantum gates, primarily aiming to expand quantum gates in the circuit using a predefined set of basic gates. For example, $\mathrm{CZ}(i,j) \equiv \mathrm{H}(j)\mathrm{CNOT}(i,j)\mathrm{H}(j)$ and $\mathrm{SWAP}(i,j) \equiv \mathrm{CNOT}(i,j)\mathrm{CNOT}(j,i)\mathrm{CNOT}(i,j)$.

S4.3 Optimization

On NISQ hardware, the goal of quantum circuit optimization is to reduce circuit depth and gate count as much as possible while preserving circuit equivalence, thereby improving overall circuit fidelity. A variety of optimization techniques have been proposed, including pattern-matching methods [12], commutation-based approaches [13], machine learning techniques [14], and algorithm-specific optimization strategies [15, 16].

However, quantum circuit optimization is not the primary focus of this study. In the current version of QSteed, we adopt only a few basic optimization techniques, such as merging parameterized quantum gates and eliminating redundant gate operations. For instance, two adjacent CNOT gates can cancel each other out, i.e., $\mathrm{CNOT}(i,j)\mathrm{CNOT}(i,j) = \mathrm{I}$. Nevertheless, QSteed still achieves excellent compilation performance through the combination of a preconstructed VQPU database and a select-then-compile workflow. Users can further implement advanced optimization components by inheriting from the `BasePass` class.

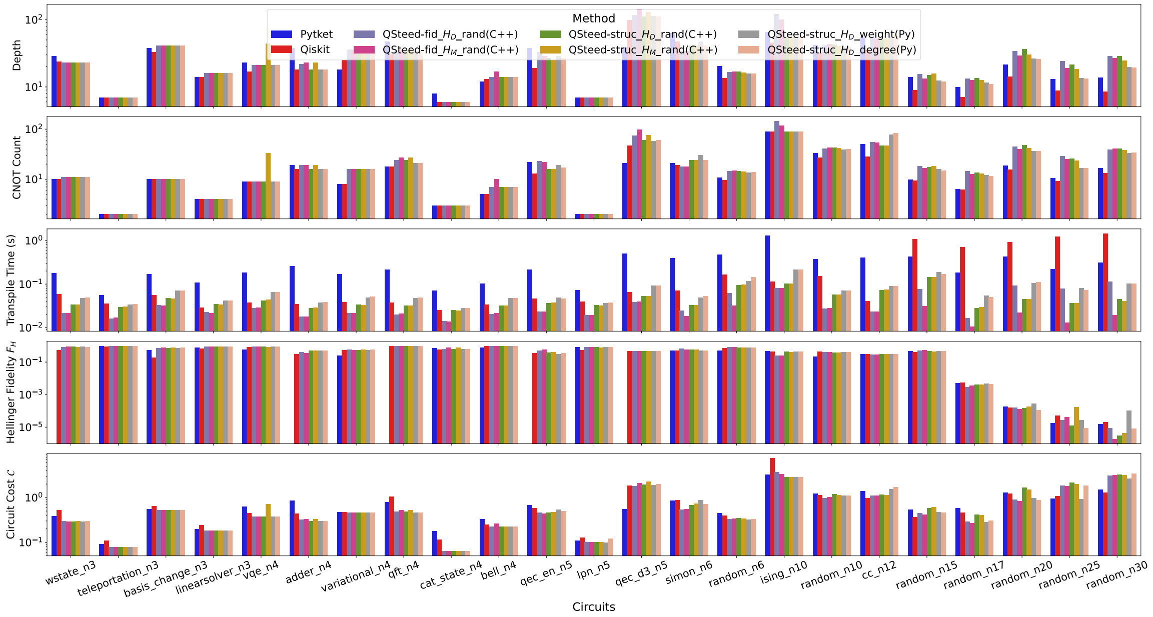

Figure S6: Performance comparison of different quantum translators on the Baihua quantum processor. The results for each benchmark circuit represent the median of 5 runs, while the results for random types are averaged over more than 50 runs of randomly generated circuits. The vertical axes, from top to bottom, represent the compiled circuit depth, the number of two-qubit CNOT gates, the transpilation time, the Hellinger fidelity F_H (higher is better), and the circuit cost function $\mathcal{C}$ (lower is better). Except for the random circuits, the circuit names on the horizontal axis are derived from the QASMBench benchmarking suite [18].

S4.4 Parameter Tuning

This component specializes in compiling parameterized quantum circuits for variational algorithms like VQE and QAOA. By integrating Pyquafu's auto-differentiation framework [17], it seamlessly handles hybrid circuits containing both variational (e.g., $\mathrm{RZZ}(i, j, \theta)$) and fixed parameters (e.g., $\mathrm{RZZ}(i, j, \pi)$). The compiler automatically optimizes parameterized gate sequences through algebraic simplification, such as reducing $\mathrm{RZZ}(i, j, \theta)\,\mathrm{RZZ}(i, j, \pi)$ to $\mathrm{CNOT}(i, j)\,\mathrm{RZ}(j, \theta + \pi)\,\mathrm{CNOT}(i, j)$, ensuring efficient circuit execution while preserving variational semantics.

S5 Performance Comparisons

This section provides further details on the compilation performance comparison among QSteed, Qiskit, and Pytket. All classical computations were carried out on a workstation equipped with an 8-core Apple M1 Pro CPU and 16 GB of memory. QSteed implements a diverse array of compilation strategies, which are denoted as QSteed-x_y_z. In this nomenclature: The variable x represents the VQPU selection strategy. Specifically, `fid` corresponds to the fidelity-first strategy, while `struc` represents the structure-first strategy. The variable y designates the heuristic function employed in the SABRE algorithm. The variable z indicates the initial layout selection for the SABRE algorithm.

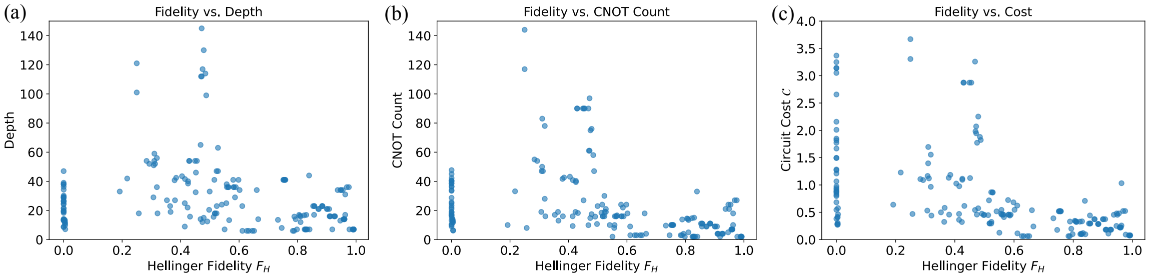

Figure S7: Negative correlation between Hellinger fidelity and circuit-complexity metrics. Scatter plots show the relationship between Hellinger fidelity F_H and (a) circuit depth, (b) two-qubit (CNOT) gate count, and (c) the composite circuit cost for the same dataset used in Figure S6. All three panels exhibit a statistically significant negative monotonic trend: Spearman rank correlations are $\rho_{\text{depth}} = -0.288$, $\rho_{\text{count}} = -0.576$, and $\rho_{\text{cost}} = -0.686$, with $p < 10^{-4}$ in every case. These results indicate that higher-fidelity circuits tend to be shallower, require fewer CNOT gates, and incur lower circuit cost. Because the circuit cost metric $\mathcal{C}$ yields the strongest (most negative) correlation with fidelity, we adopt $\mathcal{C}$ as a proxy for the expected circuit fidelity in the main text.

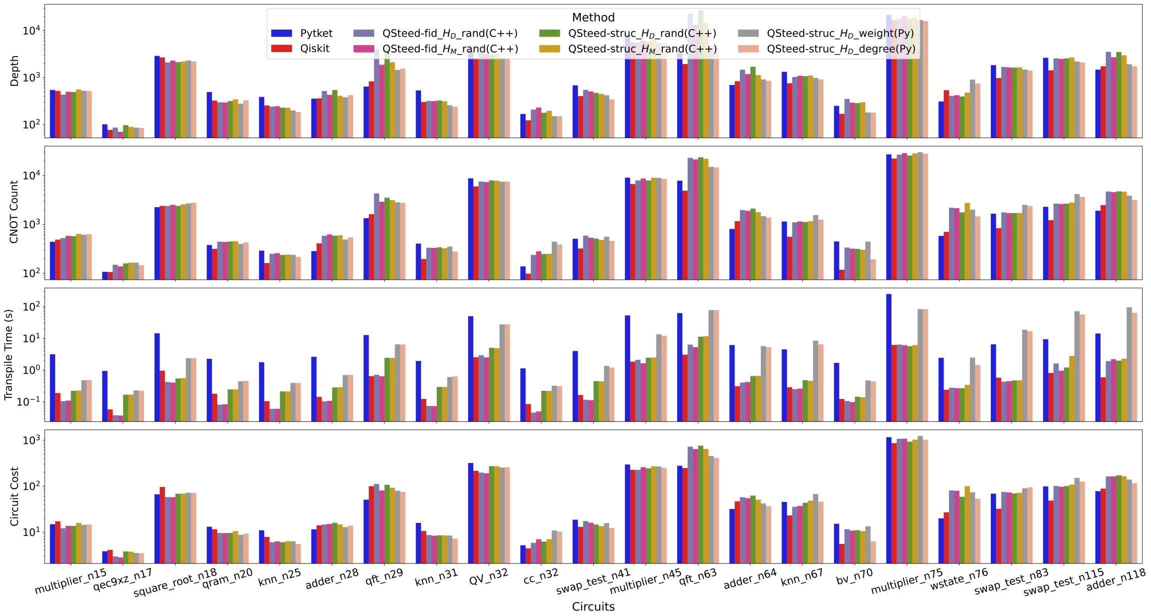

Figure S8: Due to the impact of quantum hardware noise, the sampling results of large-scale quantum circuits are often unreliable. Moreover, obtaining ideal computational results for such circuits from simulators remains challenging, making the calculation of F_H infeasible. Therefore, instead of running the circuits compiled by the three compilers on real quantum machines, we simulated and calculated the fidelity of each benchmark circuit based on the parameter information of the Baihua chip. The results for each benchmark circuit represent the median of 5 runs. The vertical axes, from top to bottom, represent the compiled circuit depth, the number of two-qubit CNOT gates, the transpilation time, and the circuit cost function $\mathcal{C}$ (lower is better). The circuit names (trailing number denotes qubits) on the horizontal axis are derived from the QASMBench benchmarking suite [18].

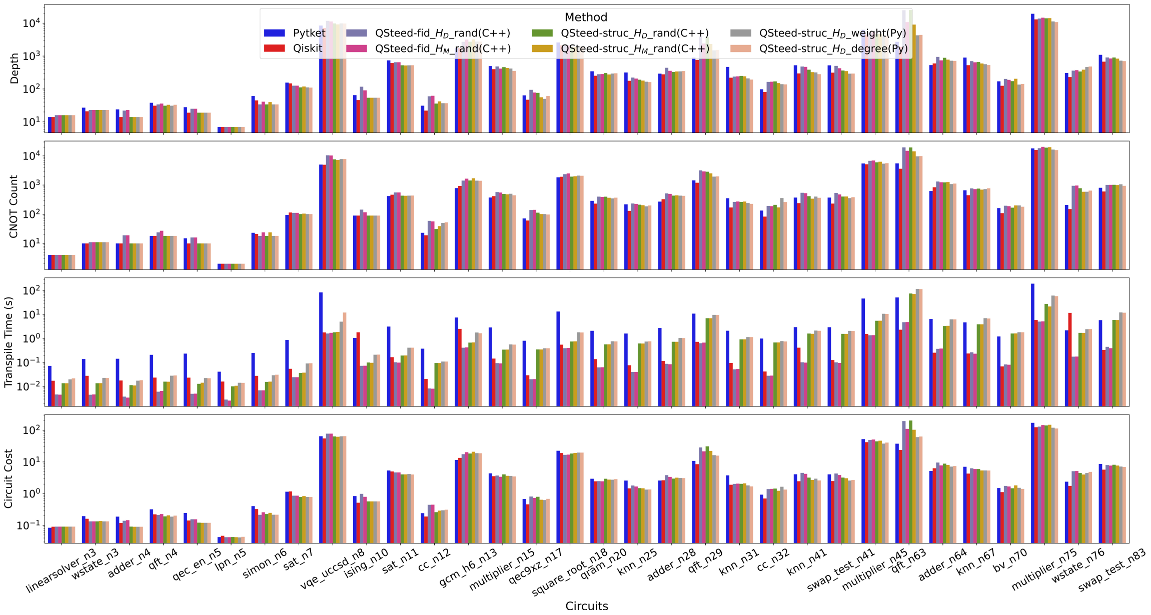

Figure S9: Performance comparison of various quantum translators through the emulation of the Willow quantum processor. The chip parameter information is sourced from [19]. The results for each benchmark circuit represent the median of 5 runs. The vertical axes, from top to bottom, represent the compiled circuit depth, the number of two-qubit CNOT gates, the transpilation time, and the circuit cost function $\mathcal{C}$ (lower is better). The circuit names (trailing number denotes qubits) on the horizontal axis are derived from the QASMBench benchmarking suite [18].

Here, `rand` stands for random initialization, `degree` represents degree initialization, and `weight` denotes weight initialization. QSteed was originally implemented entirely in Python. Because the qubit-mapping and DAG passes were among the more time-consuming components of the system, we subsequently re-implemented them in C++ to improve performance. In the subsequent descriptions of QSteed compilation strategies, the suffix `Py` refers to results obtained with the pure-Python implementation, whereas `C++` denotes runs in which the critical passes are accelerated by C++.

S5.1 Experimental Results and Fidelity–Cost Relationship

Figure S6 presents the benchmarking results obtained on the Baihua processor. We first retrieved the latest calibration data for the chip from the cloud and verified its operational status using ErrorGnoMark [20], confirming that it was functioning within a reasonable regime. Based on the calibration data, we constructed a VQPU database locally on a personal computer. Each benchmark circuit was then compiled using different toolchains, and the resulting circuits were executed on the cloud via `QuarkStudio` [21] to obtain real hardware sampling results, from which Hellinger fidelity F_H was computed. We also computed the cost $\mathcal{C}$ using the same set of circuits. All results are summarized in Figure S6.

Figure S7 visualizes the correlations between circuit fidelity and key compilation metrics using scatter plots. The plots show that circuit depth, CNOT gate count, and overall circuit cost $\mathcal{C}$ are all negatively correlated with Hellinger fidelity F_H. To quantify the strength of these relationships, we compute the Spearman rank correlation coefficients, which are $\rho_{\text{depth}} = -0.546$, $\rho_{\text{count}} = -0.633$, and $\rho_{\text{cost}} = -0.678$, with $p < 10^{-4}$ in all cases. These results indicate that higher-fidelity circuits tend to be shallower, require fewer CNOT gates, and incur lower circuit cost. Among the three metrics, the circuit cost $\mathcal{C}$ exhibits the strongest (most negative) correlation with fidelity, which supports our use of $\mathcal{C}$ as a surrogate predictor of circuit fidelity throughout the main text.

S5.2 Simulation results

FigureS8 and FigureS9 show simulation results on the Baihua and Google's Willow processors, respectively, as supplements to the main discussion.

S5.3 System Stability under Hardware Calibration Dynamics

Due to environmental factors, quantum hardware may experience calibration parameter drift. To evaluate the robustness of our compilation system under such conditions, we selected the Baihua processor as the target backend (which is typically recalibrated once per day) and developed an automated script that executed tasks every two hours over two consecutive days. Specifically, we compiled and executed the vqe_n4 and cc_n4 circuits every two hours to monitor the stability of the virtualization strategy. As shown in Figure S10 and S11, the compilation performance remained nearly constant during this period.

The VQPU database in QSteed is dynamically linked to the hardware calibration data. When the backend hardware undergoes recalibration, the updated parameters are pushed to the virtualization

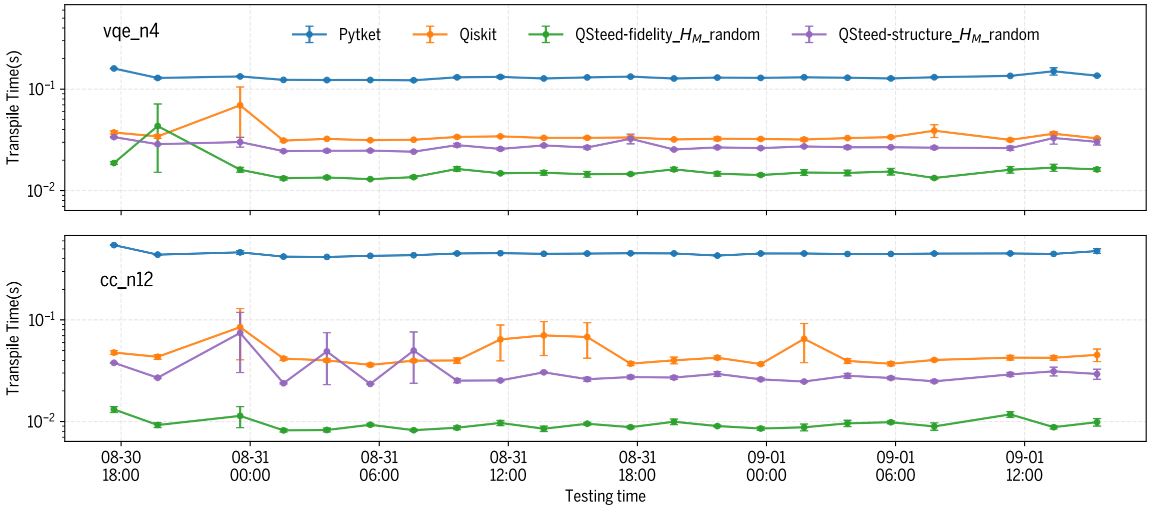

Figure S10: Between August 30 and September 1, 2025, circuit tasks were executed every two hours to monitor variations in compilation time. The chip is typically recalibrated once per day.

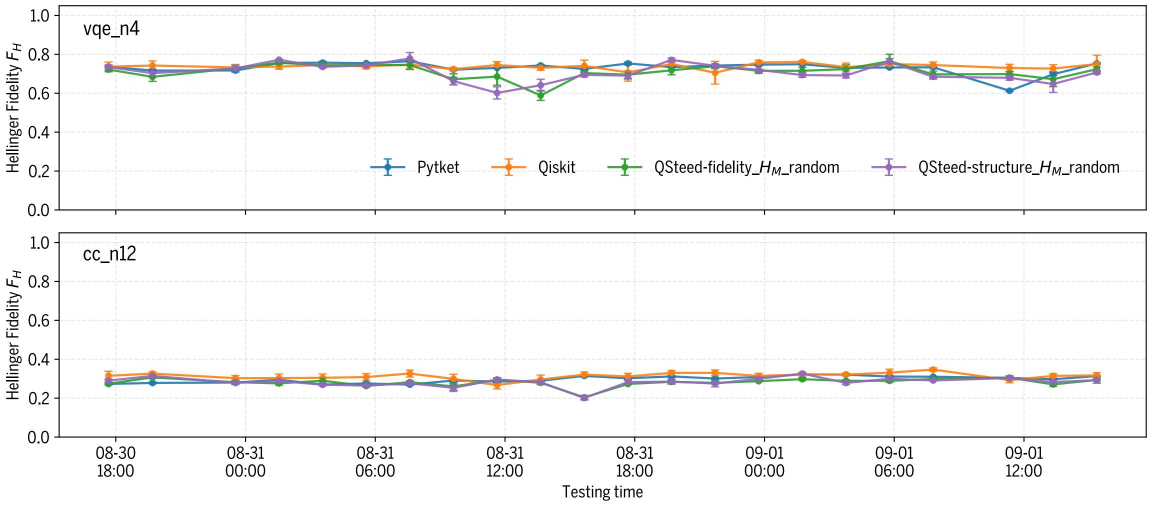

Figure S11: Between August 30 and September 1, 2025, circuit tasks were executed every two hours to monitor variations in compiled circuit execution fidelity. The chip is typically recalibrated once per day.

manager, which refreshes the VQPU database accordingly. It is important to note, however, that while the VQPU database is dynamically synchronized with calibration data, the system cannot detect drift if the hardware itself is not recalibrated in time. This represents a current limitation of our approach. Nevertheless, such issues cannot be fully addressed at the compilation level alone and are beyond the scope of this study. Future improvements could be achieved by integrating the compiler with chip benchmarking and automated calibration systems. For example, fast benchmarking routines (e.g., parallel measurements of GHZ states on two-qubit gates to monitor qubit stability) could be executed periodically, and once qubit drift is detected, an automatic calibration procedure could be triggered to update the hardware parameters.

References

[1] BAQIS-Quantum. Qsteed, 2024. https://github.com/BAQIS-Quantum/qsteed.

[2] Nino Shervashidze, Pascal Schweitzer, Erik Jan van Leeuwen, Kurt Mehlhorn, and Karsten M. Borgwardt. Weisfeiler-lehman graph kernels. *Journal of Machine Learning Research*, 12(77):2539–2561, 2011.

[3] Mikko Möttönen and Juha J Vartiainen. Decompositions of general quantum gates. *Trends in quantum computing research*, page 149, 2006.

[4] V.V. Shende, S.S. Bullock, and I.L. Markov. Synthesis of quantum-logic circuits. *IEEE Transactions on Computer-Aided Design of Integrated Circuits and Systems*, 25(6):1000–1010, 2006.

[5] Mikko Möttönen, Juha J. Vartiainen, Ville Bergholm, and Martti M. Salomaa. Quantum circuits for general multiqubit gates. *Phys. Rev. Lett.*, 93:130502, September 2004.

[6] Marc G. Davis, Ethan Smith, Ana Tudor, Koushik Sen, Irfan Siddiqi, and Costin Iancu. Towards optimal topology aware quantum circuit synthesis. In *2020 IEEE International Conference on Quantum Computing and Engineering (QCE)*, pages 223–234, 2020.

[7] Liam Madden and Andrea Simonetto. Best approximate quantum compiling problems. *ACM Transactions on Quantum Computing*, 3(2), March 2022.

[8] Yuan-Hang Zhang, Pei-Lin Zheng, Yi Zhang, and Dong-Ling Deng. Topological quantum compiling with reinforcement learning. *Phys. Rev. Lett.*, 125:170501, October 2020.

[9] Lorenzo Moro, Matteo GA Paris, Marcello Restelli, and Enrico Prati. Quantum compiling by deep reinforcement learning. *Communications Physics*, 4(1):178, 2021.

[10] Adriano Barenco, Charles H. Bennett, Richard Cleve, David P. DiVincenzo, Norman Margolus, Peter Shor, Tycho Sleator, John A. Smolin, and Harald Weinfurter. Elementary gates for quantum computation. *Phys. Rev. A*, 52:3457–3467, November 1995.

[11] Farrokh Vatan and Colin Williams. Optimal quantum circuits for general two-qubit gates. *Phys. Rev. A*, 69:032315, March 2004.

[12] Raban Iten, Romain Moyard, Tony Metger, David Sutter, and Stefan Woerner. Exact and practical pattern matching for quantum circuit optimization. *ACM Transactions on Quantum Computing*, 3(1), January 2022.

[13] Toshinari Itoko, Rudy Raymond, Takashi Imamichi, and Atsushi Matsuo. Optimization of quantum circuit mapping using gate transformation and commutation. *Integration*, 70:43–50, 2020.

[14] Zikun Li, Jinjun Peng, Yixuan Mei, Sina Lin, Yi Wu, Oded Padon, and Zhihao Jia. Quarl: A learning-based quantum circuit optimizer. *Proceedings of the ACM on Programming Languages*, 8(OOPSLA1):555–582, 2024.

[15] Lingling Lao and Dan E. Browne. 2qan: A quantum compiler for 2-local qubit hamiltonian simulation algorithms. In *Proceedings of the 49th Annual International Symposium on Computer Architecture*, ISCA '22, page 351–365, New York, NY, USA, 2022. Association for Computing Machinery.

[16] Hong-Ze Xu, Wei-Feng Zhuang, Zheng-An Wang, Kai-Xuan Huang, Yun-Hao Shi, Wei-Guo Ma, Tian-Ming Li, Chi-Tong Chen, Kai Xu, Yu-Long Feng, et al. Quafu-qcover: Explore combinatorial optimization problems on cloud-based quantum computers. *Chinese Physics B*, 33(5):050302, 2024.

[17] Quafu quantum cloud computing cluster. https://quafu.baqis.ac.cn/.

[18] Ang Li, Samuel Stein, Sriram Krishnamoorthy, and James Ang. Qasmbench: A low-level quantum benchmark suite for nisq evaluation and simulation. *ACM Transactions on Quantum Computing*, 4(2), February 2023.

[19] Rajeev Acharya, Laleh Aghababaie-Beni, Igor Aleiner, Trond I Andersen, Markus Ansmann, Frank Arute, Kunal Arya, Abraham Asfaw, Nikita Astrakhantsev, Juan Atalaya, et al. Quantum error correction below the surface code threshold. *Nature*, December 2024.

[20] BAQIS-Quantum. Errorgnomark, 2025. https://github.com/BAQIS-Quantum/ErrorGnoMark.

[21] Quarkstudio, 2025. https://quarkstudio.readthedocs.io/en/latest.
